# Assessment of knowledge and practices of additive manufacturing in dentistry among university teaching faculty in Saudi Arabia

**DOI:** 10.1186/s12903-024-04037-8

**Published:** 2024-02-24

**Authors:** Khalid K. Alanazi, Abdulaziz A. Alzaid, Adel Alotaibi, Nora Almehisni, Ghida Alzahrani, Khalid Gufran

**Affiliations:** 1 Conservative Dental Science Department, College of Dentistry, Prince Sattam bin Abdulaziz University, Alkharj, 11942 Saudi Arabia; 2 Restorative and Prosthetic Dental Sciences Department, College of Dentistry, King Saud Bin Abdulaziz University for Health Sciences, Riyadh 11426, Saudi Arabia; 3 King Abdullah International Medical Research Center, Ministry of National Guard-Health Affairs, Riyadh 11481, Saudi Arabia; 4 Preventive Dental Science Department, College of Dentistry, King Saud Bin Abdulaziz University for Health Sciences, Riyadh 11426, Saudi Arabia; 5 Dental Intern, College of Dentistry, King Saud Bin Abdulaziz University for Health Sciences, Riyadh 11426, Saudi Arabia; 6 Department of Preventive Dental Sciences, College of Dentistry, Prince Sattam bin Abdulaziz University, Alkharj, 11942 Saudi Arabia

**Keywords:** 3D printing, CAD/CAM, Dental curriculum, Dental education, Digital dentistry

## Abstract

**Background:**

In recent era, digitalization in the dental sciences has been observed in wide ranges. This cross-sectional study aimed to assess knowledge and practice of additive manufacturing (AM) in dentistry among university teaching faculty in Saudi Arabia.

**Methods:**

A questionnaire was prepared and validated to distribute to the different dental colleges in Saudi Arabia. The questionnaire was divided into three parts: demographic information, knowledge and practices of AM among the dental teaching faculty. After receiving all the responses, descriptive statistics were used for the frequency distribution of all the responses.

**Results:**

A total of 367 responses were received from the different faculty members. Most of the participants were male (67.30%), holding assistant professor (52.50%) positions in the field of prosthodontics (23.40%). In terms of knowledge, even though most of the participants were aware of AM (64.30%); however, do not understand the AM techniques (33.50). Moreover, 71.90% of the participants had no experience working with AM and only 13.60% of participants used AM in their respective dental colleges.

**Conclusion:**

AM techniques are not commonly used in the field of dentistry in Saudi Arabia; therefore, more platforms should have created to enhance the knowledge and practice of AM in the current population.

**Supplementary Information:**

The online version contains supplementary material available at 10.1186/s12903-024-04037-8.

## Background

In recent years, digitalization in the field of dentistry has been immensely observed. Many digital applications have evolved, and the advancement of these technologies makes dental practice easier and more efficient. Additive manufacturing (AM) is one of the advanced digital technologies which facilitates work in dental laboratories. AM also known as three-dimensional (3D) printing implicates the addition of materials layer upon layer in order to construct a structure or an object by using advanced scanning and imaging with computer-aided design/ computer-aided manufacturing (CAD/CAM) technologies [1–3].

AM techniques could be applied in different fields of dentistry. Due to the advantages of the AM techniques, optimum results could be obtained in constructing digital models, digital impressions, face bows, orthodontic aligners, and prostheses including veneers, bridges, crowns, and laminates. virtual articulators. Moreover, tissue scaffolds for soft and hard tissue, implants in the maxillofacial region could be 3D printed which plays an important role in implantology and craniofacial reconstruction [3–7]. 3D dental impression using AM techniques is less time-consuming and cost-effective compared to the traditional impression technique. The issues with traditional impressions such as proper tray selection, distortion of impression, trimming error, polymerization shrinkage, and soft tissue management could easily be eliminated with the AM techniques [8].

AM techniques easily use a variety of materials such as ceramics, polymers, and metals to fabricate customized feasible treatments for the patients. It could create a customized implant for a deformed jawbone. Moreover, partial and complete prosthetic dentures along with crown and restoration using the AM technique facilitate better fitting to the patients.

It also reported that maxillofacial prostheses constructed with AM results cut down the surgery time [9, 10]. Preparing a single-tooth crown is relatively simple and clinicians could fabricate it in one day using the AM techniques. These advanced technologies increase the efficiency and productivity of dental practices. Even though the AM technique or 3D printing is available since 1986 [11–13], its usage of this is still limited due to operator calibration, cost-efficiency, and material compatibility [14]. Therefore, the knowledge and practices of AM in dental practices were assessed in previous studies which showed only a few percentages of dentists experienced or using AM techniques in their daily practices [15–18]. It is important to ascertain the knowledge and practices of such advanced technologies in specific populations to identify the gaps which enable com-prehending future action in planning and applying AM techniques among dentists to improve treatment outcomes for patients. Hence, this study aimed to assess the knowledge and practices of AM in dentistry among university teaching faculties in Saudi Arabia.

## Methods

The current descriptive cross-sectional study was conducted in the College of Dentistry, Prince Sattam Bin Abdulaziz University. The standing committee of bioethics research (SCBR) of Prince Sattam bin Abdulaziz University approved this study protocol (SCBR-011-2023). Moreover, the study was conducted according to the guidelines of the Declaration of Helsinki.

This study aimed to evaluate the knowledge and practices of using additive manufacturing among the dental teaching faculty in Saudi Arabia. An original questionnaire was prepared to distribute to the different dental colleges in Saudi Arabia. The questionnaire was divided into three parts. The first part contained the demographic information of participants with four questions. Parts two and three are confined to eight and 11 questions about the knowledge and practices of 3D printing among the dental teaching faculty, respectively (Supplementary document). The content of the questionnaire was validated by a pilot study among 40 professionals from Prince Sattam bin Abdulaziz University with content and face validity. The content and face validity ratio were 0.750 and 0.920, respectively.

After attaining the official approval of the study, authorities of Prince Sattam bin Abdulaziz University distributed the questionnaire to the different dental colleges in Saudi Arabia through the online survey website ‘google forms’. Participants were reached out using the official email requested to distribute the form among colleagues through convenient social media platforms and emails. Informed consent was obtained from all the participants who agreed to participate in this study. The study was explained and ensured confidentiality to all the participants. A brief introduction about the study was given and filling the questionnaire was considered as giving consent to participate in this study. All the responses were collected in 3 months’ time frame.

### Statistical analyses

Statistical analyses were performed using the statistical package for Social Science (SPSS) version 27 (IBM, Armonk, USA). Descriptive statistics were used for the frequency distribution of all the responses.

## Results

A total of 367 responses (93% of the responses received against number of questionnaires administered) were collected from the different faculty members of dental colleges in Saudi Arabia. The questionnaire was divided into three parts: demographic information, knowledge, and practices of 3D printing among dental faculties. The frequency distribution of the demographic information of the participants showed that 67.30% of participants were male faculty members. The majority of the participants were assistant professors (52.50%) and from the specialty of prosthodontic dentistry (23.40%). Two faculty members have more than one specialty. Moreover, most participants were from King Saud bin Abdulaziz University for Health Sciences (12.30%), and no response was obtained from Mustaqbal University and Alfarabi University. The frequency distribution of the demographic information of the participants was presented in Table 1.

**Table 1:** Demographic information of participated teaching faculty

Variables	Frequency	Percentages (%)
Gender		
Male	247	67.30
Female	120	32.70
Position		
Professor	23	6.30
Associate professor	48	13.10
Assistant Professor	193	52.50
Lecturer	80	21.80
Teaching Assistant	23	6.30
Specialty		
GP Dentist	12	3.30
Restorative dentistry	59	16.10
Prosthodontics	86	23.40
Orthodontics	33	9.00
Periodontics	35	9.50
Endodontics	26	7.10
Oral and maxillofacial surgery	19	5.20
Oral and maxillofacial pathology	21	5.70
Oral medicine	15	4.10
Pediatric Dentistry	27	7.40
Oral radiology	11	3.00
More than one	2	0.60
Other	20	5.40
University		
King Saud University	21	5.70
King Saud Bin Abdulaziz University for Health Sciences	45	12.30
Prince Sattam Bin Abdulaziz University	44	12.00
Princess Nourah Bint Abdulrahman University	12	3.30
Majmaah University	8	2.20
Riyadh Elm University	12	3.30
Dar Al Uloom University	8	2.20
Vision Colleges	9	2.50
King Abdulaziz University	13	3.50
King Khalid University	11	3.00
Taibah University	12	3.00
Um Alqura University	10	2.70
King Faisal University	10	2.70
Qassim University	11	3.00
Mustaqbal University	0	0.00
Taif University	27	7.40
University of Hail	10	2.70
Jazan University	36	9.80
Jouf University	20	5.40
Baha University	11	3.00
Najran University	9	2.50
Alfarabi University	0	0.00
Ibn Sena University	9	2.50
Batterjee Medical College	8	2.20
Imam Abdulrahman bin Faisal University	11	3.00

Frequency distribution of the knowledge of AM among the dental faculties showed that the majority of the participants were aware of the usage of AM in dentistry (64.30%) as well as the fields other than dentistry (54.50%). However, most participants not understood (33.50%) the AM technology. A total of 34.30% of participants did not obtain any information in their study period regarding additive manufacturing which is the major frequency distribution and 0.05% of participants acquired information about AM during the fellowship program. Even though the majority of the participants (62.10%) are not aware of the different additive manufacturing techniques, a total of 24.30% of participants are familiar with more than one AM technology. Most of the participants (78.20%) are not mindful of the facilities of additive manufacturing techniques provided by their university and 59.70% of faculty members are not conscious about acquiring AM in their respective universities within the next two years. The frequency distribution of the knowledge of AM of the participants was presented in Table 2.

**Table 2:** Knowledge of additive manufacturing in dentistry among university teaching faculty in Saudi Arabia

Questionnaire	Frequency	Percentages (%)
Q1		
Yes	236	64.30
No	131	35.70
Q2		
Yes	200	54.50
No	167	45.50
Q3		
Well understood	29	7.90
Good understanding	97	26.40
Fairly understood	118	32.20
Not understood	123	33.50
Q4		
Undergrad education	6	1.60
Postgrad education	68	18.50
Fellowship	2	0.50
Seminars	7	1.90
Workshops	9	2.50
Continuous education lectures	43	11.70
Others	27	7.40
Not obtained	126	34.30
More than one	79	21.50
Q5		
Yes	139	37.90
No	228	62.10
Q6		
Stereolithography (SLA)	22	6.00
Digital light processing (DLP)	10	2.70
Selective laser sintering (SLS)	7	1.90
Selective laser melting (SLM)	6	1.60
Direct metal laser sintering (DMLS)	1	0.30
Direct deposition modeling/jetting	6	1.60
Other	36	9.80
More than one	89	24.30
Q7		
Yes	80	21.80
No	287	78.20
Q8		
Yes	64	17.40
No	219	59.70
Skipped	84	22.90

Q1; Are you aware of the use of additive manufacturing in dentistry?, Q2; Are you aware of additive manufacturing in fields other than dentistry?, Q3; How would you describe your comprehension of additive manufacturing technology? Q4; How did you obtain information about additive manufacturing in dentistry?, Q5; Are you aware of different additive manufacturing techniques?; Q6; If yes, please select additive manufacturing techniques you’re familiar with Q7; Are you aware of any additive manufacturing facility in your university?, Q8; If no, are you aware of any plans to acquire such facility in the next 2 years? (Please skip this question if you answered yes in the previous question)

Frequency distribution of the practices of AM among dental faculties showed that 71.90% of the participants had no experience working with AM and only 13.60% of participants used AM in their respective dental colleges. Participants who had experience with AM technique very often use AM (7.60%) in their practice in different branches of dentistry (14.30%). The majority of the participants do not use AM techniques in their private practice (83.90%). Even those who have AM techniques in their private practice setting occasionally AM used (8.70%). Most of the participants used the resin materials (19.10%) as AM. The curriculum of the respective university covers 33.30% of AM in dentistry and most of the participants (18.80%) believe that it needs to be improved. Even though the curriculum does not cover the AM techniques in dentistry, only 31.10% of participants ensure the plans to incorporate it in the curriculum in the next 2 years. The frequency distribution of the practices of AM of the participants was presented in Table 3.

**Table 3:** Practices of additive manufacturing in dentistry among university teaching faculty in Saudi Arabia

Questionnaire	Frequency	Percentages (%)
Q1		
Yes	103	28.10
No	264	71.90
Q2		
Yes	50	13.60
No	317	86.40
Q3		
An integral part of my dental practice	12	3.30
Regular bases	18	4.90
Occasionally	17	4.60
Not often	28	7.60
Only for teaching purposes	20	5.40
Skipped	272	74.10
Q4		
Diagnostic casts and models	2	0.50
Fixed prosthodontics	11	3.00
Removable prosthodontics	0	0.00
Surgical guides for implant placement	7	1.90
Radiographic stents	2	0.50
Occlusal appliances	1	0.30
Orthodontic aligners	4	1.10
Anatomical models for pre-surgical assessment, planning, and training	0	0.00
Scaffold for tissue engineering	1	0.30
Other	15	4.10
More than one	52	14.30
Skipped	272	74.10
Q5		
Yes	59	16.10
No	308	83.90
Q6		
An integral part of my dental practice	8	2.20
Regular bases	22	6.00
Occasionally	32	8.70
Not often	32	8.70
Skipped	273	74.40
Q7		
Diagnostic casts and models	3	0.80
Fixed prosthodontics	9	2.50
Removable prosthodontics	0	0.00
Surgical guides for implant placement	8	2.20
Radiographic stents	3	0.80
Occlusal appliances	1	0.30
Orthodontic aligners	11	3.00
Anatomical models for pre-surgical assessment, planning, and training	1	0.3
Scaffold for tissue engineering	2	0.50
Other	15	4.10
More than one	42	11.80
Skipped	272	74.10
Q8		
Resin	70	19.10
Metal	7	1.90
Ceramics	23	6.30
More than one	30	8.20
Skipped	237	64.50
Q9		
Yes	115	31.30
No	252	68.70
Q10		
Overly covered	4	1.10
Well covered	15	4.10
Sufficiently covered	45	12.30
Needs Improvement	69	18.80
Skipped	234	63.80
Q11		
Yes	114	31.10
No	136	37.10
Skipped	116	31.60

Q1; Have you had any experience working with additive manufacturing?, Q2; Do you use additive manufacturing in your dental college? Q3; If yes, how would you describe your practice? (Please skip this question if you answered no in the previous question), Q4; If yes, please select additive manufacturing application from below (Please skip this question if you answered no in the previous question), Q5; Do you use additive manufacturing in your private prac-tice?, Q6; If yes, how would you describe your practice? (Please skip this question if you answered no in the previous question), Q7; If yes, please select the additive manufacturing application form below (Please skip this question if you answered no in the previous question), Q8; If you have previously worked with additive manufacturing, what type of material have you used? Q9; Does the curriculum of your university cover additive manufacturing in dentistry?, Q10; If yes, how would you describe it (Please skip this question if you answered no in the previous question), Q11; If no, are there any plans to incorporate it in the curriculum during the next 2 years (Please skip this question if you answered yes in the previous question)

## Discussion

This current study aimed to evaluate the knowledge and practices of AM in dentistry among university teaching faculties in Saudi Arabia. There are possible fields in the medical and dental sciences to use the AM in day-to-day practice [1]. Even though the advantages of using AM, there are very limited knowledge and practices about this digital technique. Moreover, there was a scarcity of research related to the knowledge and practices of AM in the field of dentistry. Faculty members of any field who are directly engaged in the fabrication of AM are playing an important role in expanding the usage of advanced technologies. Therefore, knowledge and practices of AM among the different faculty members are imperative to distinguish.

Demographic information for the teaching faculty member is important as it con-tributes foremost roles in the usage of any AM. The demographic information of the current study showed that male faculty members predominantly participated in this study compared to female participants. Similar findings were observed in one of the previous studies which assessed the knowledge and practices of AM in terms of gender [16]. However, the opposite distribution was also observed in other studies [15, 17]. This might be due to the social standing as both studies which showed male prevalence including the current study were conducted in Saudi Arabia and in general, the male workforce in the dental sciences is more prevalent than the female [19, 20]. It showed that most of the faculty members who participated in this survey held the assistant professor rank. None of the previous studies assessed the knowledge and practices of AM specifically in teaching faculty members; therefore, direct comparison is not possible in terms of the position of the participants. Moreover, the majority of the participants were from the specialty of the prosthodontic department. A recent study on dental workforce distribution such as Saudi Arabia showed that the majority of the practitioners were general dentists followed by prosthodontists [19]. In this study, a lower number of responses were obtained from the general dentists, the reason behind this might be due to the lower numbers of general dentists appointed in the teaching faculty as teaching faculty required specialists. Since the second-highest dental workforces were from the prosthodontic department in Saudi Arabia as per Alqahtani et al. [19], it complies with the outcome of the current study. In addition, the maximum number of responses were obtained from the King Saud bin Abdulaziz University for Health Sciences which could be easily estimated as this is one the largest universities in Saudi Arabia.

In terms of knowledge of AM, the current study showed that the majority of the faculty members were aware of the use of AM in dentistry as well as other field than dentistry and this outcome is in line with the outcome of previous studies [15, 17]. However, the percentage of awareness was most observed in the study by Acharya et al. [15]. This is not an unexpected outcome as teaching faculty members are well trained in different fields of dentistry and continuously learn new technologies in order to incorporate those in the syllabus of the students to remain the curriculum updated alongside the other parts of the world. Even though the majority of the participants were aware of the AM, most of them do not understand this technology properly and did not obtain any formal information about these techniques. However, the majority who attained post-graduation were gain information about the AM techniques as a part of their post-grad study. Dhokar et al. [17] also reported in their study that post-graduate degree holders are more aware of this advanced technique. It seems that other than postgraduation study, lectures and/ or webinars are the most common way to attain knowledge about 3D painting or AM [16, 18].

When coming to the different AM techniques, most of the participants were not aware of the different types of techniques and those who were aware of this technology recognize more than one technique. Moreover, Stereolithography is the most common technique of which the majority of the participants were aware, and a similar consequence was also observed in the study by Acharya et al. [15]. Surprisingly, the majority of the faculty members aware of the facilities of their respective universities nor any plans to acquire such facilities in the next 2 years. Only awareness of the specific technique is not sufficient, proper comprehension of certain advanced techniques is important to encompass in daily practice to attain the utmost advantages. Due to the limited knowledge and under-standing of the AM in the current population, the exchange of information, experience, and training should be conducted via social media, webinars, and workshops.

In terms of practicing the AM, the majority of the participants stated no experience of working with additive manufacturing. According to the outcome of frequency distribution, the greatest number of the participants do not use AM technique in their respective dental schools and opted to skip most of the questions in the practice part of the questionnaire. Even those who opt to use the AM in dental schools, seldom use the technique. None of the participants use AM technique in removable prosthodontic and Anatomical models for pre-surgical assessment, planning, and training. However, previous studies reported that the fabrication of AM before a surgical procedure makes the surgery more accurate [18, 21–23]. Resin is the most used material for AM in the current population. A survey from Acharya et al. [15] also showed that resin is more compatible with using AM compared to the other materials. Not many universities cover the AM technique in the school curriculum; even the university which covers this technique needs to be improved as per the participants. This explained why the majority of the participants did not receive any information about AM technology in their graduate study as most of the universities do not cover this topic. Moreover, it assumed from the responses that there is no plan to incorporate this topic into the curriculum for the next two years.

Even though AM techniques have been introduced many years before, the current study similar to some previous studies [15–18] showed that the usage of AM technology is not commonly used to date. The current study was conducted among the teaching faculties only; however, the AM techniques are mostly used by dental technicians. Therefore, surveys on different laboratory and dental technicians could have provided different outcomes. Moreover, years of experience and age group of the participants were not taken into consideration in this study. Previous studies showed that younger dental practitioners are more aware of AM techniques compared to older practitioners [15–17]. Moreover, participants with experience from two to five years are more prevalently aware of the AM techniques [15]. In addition, the uses of AM techniques vary in different fields of dentistry. Even though AM used in many fields of the dentistry, the availability of the materials and adopting the AM practices in the regular basis is challenging. Using AM requires special training, and it is not widely used around the world. Saudi Arabia invest a lot in the healthcare budget and improving the healthcare system is one of the main areas of ‘Vision 2030’ [19]. Therefore, the latest technologies in different dental fields including the AM are nationwide available in every university in Saudi Arabia. Moreover, appropriate training opportunities in adopting AM are also available in dental schools and colleges. Therefore, the teaching faculties of the Saudi Arabia should have well informed and able to utilize the facilities of AM techniques. However, the outcome of the current study did not fully satisfy in terms of the knowledge and usage of AM among the current population compared to the resources available which need to be monitored. Some fields or specialties use advanced techniques more than others such as prosthodontic field of dentistry uses more advanced technique compared to the other field of dentistry due to the availability of the materials. Therefore, it would also provide more insight if the questionnaire was focused on specific fields and faculty members of the current population and later could compare the outcome. Hence, future study is recommended to eliminate all the limitations in the current study.

## Conclusion

Even though AM techniques gaining popularity in the field of dentistry, the usage of AM is not as popular as the other part of the world in the current population. Therefore, more platforms should be created where dentists in the current population gain more knowledge about different techniques of AM and implement them in day-to-day dental practice. Moreover, the university curriculum should include AM; hence, more knowledge and practices of AM in dentistry would inaugurate.

### Supplementary Information

**Supplementary Material 1.**

## Data Availability

The datasets used and/or analyzed during the current study are available from the corresponding author on reasonable request.

## Abbreviations

AM: Additive manufacturing
3D: Three-dimensional
CAD/CAM: Computer-aided design/ Computer-aided manufacturing
SCBR: Standing committee of bioethics research
SPSS: Statistical package for Social Science
%: Percentage
