# Supplementary material for: Assessment of knowledge and practices of additive manufacturing in dentistry among university teaching faculty in Saudi Arabia
Source: BMC Oral Health. 2024 Feb 24;24:271. doi: 10.1186/s12903-024-04037-8 (PMC10893747; doi:10.1186/s12903-024-04037-8)
Supplement: Supplementary file 1 — Supplementary Material 1. [file 12903_2024_4037_MOESM1_ESM.pdf]

# Assessment of Knowledge and Practices of Additive Manufacturing in Dentistry among University Teaching Faculty in Saudi Arabia

1. **Gender:**
   A. Male
   B. Female

2. **Position:**
   A. Professor
   B. Associate Professor
   C. Assistant Professor
   D. Lecturer
   E. Teaching Assistant

3. **Specialty:**
   A. GP Dentist
   B. Advanced General Dentist
   C. Restorative Dentistry
   D. Prosthodontics
   E. Orthodontics
   F. Periodontics
   G. Endodontics
   H. Oral and Maxillofacial Surgery
   I. Oral and Maxillofacial Pathology
   J. Oral Medicine
   K. Pediatric Dentistry
   L. Oral Radiology
   M. Other

4. **University:**
   A. King Saud University
   B. King Saud Bin Abdulaziz University for Health Sciences
   C. Prince Sattam Bin Abdulaziz University
   D. Princess Nourah Bint Abdulrahman University
   E. Majmaah University
   F. Riyadh Elm University
   G. Dar Al Uloom University
   H. Vision Colleges
   I. King Abdulaziz University
   J. King Khalid University

K. Taibah University
L. Um Alqura University
M. King Faisal University
N. Qassim University
O. Mustaqbal University
P. Taif University
Q. University of Hail
R. Jazan University
S. Jouf University
T. Baha University
U. Najran University
V. Ibn Sena University
W. Batterjee Medical College
X. Imam Abdulrahman bin Faisal University

5. **Are you aware about the use of additive manufacturing in dentistry?**
   A. Yes
   B. No

6. **Are you aware of additive manufacturing in fields other than dentistry?**
   A. Yes
   B. No

7. **How would you describe your comprehension of additive manufacturing technology?**
   A. Well understood
   B. Good understanding
   C. Fairly understood
   D. Not understood

8. **How did you obtain information about additive manufacturing in dentistry?**
   A. Undergrad education
   B. Post grad education
   C. Fellowship

D. Seminars
E. Workshops
F. Continuous education lectures
G. Others
H. Not obtained

**9. Are you aware about different additive manufacturing techniques?**

A. Yes
B. No

**10. If yes, please select additive manufacturing techniques you're familiar with ?**

A. Stereolithography (SLA)
B. Digital light processing (DLP)
C. Selective laser sintering (SLS)
D. Selective laser melting (SLM)
E. Direct metal laser sintering (DMLS)
F. Direct deposition modeling/jetting
G. Other

**11. Are you aware of any additive manufacturing facility in your university?**

A. Yes
B. No

**12. If no, are you aware of any plans to acquire such facility in the next 2 years? (please skip this question if you answered YES in the previous question)**

A. Yes
B. No

**13. Have you had any experience of working with additive manufacturing?**

A. Yes
B. No

**14. Do you use additive manufacturing in your dental college?**

A. Yes
B. No

**15. If yes, how would you describe your practice?**
**( please skip this question if you answered NO in the previous question)**

A. Integral part of my dental practice
B. Regular bases
C. Occasionally
D. Not often
E. Only for teaching purposes

**16. If yes, please select additive manufacturing application from below:**
**(please skip this question if you answered NO in question 14)**

A. Diagnostic casts and models
B. Fixed prosthodontics
C. Removable prosthodontics
D. Surgical guides for implant placement
E. Radiographic stents
F. Occlusal appliances
G. Orthodontic aligners
H. Anatomical models for pre-surgical assessment, planning and training
I. Maxillofacial surgery
J. Scaffold for tissue engineering
K. Other

**17. Do you use additive manufacturing in your private practice?**

A. Yes
B. No

**18. If yes, how would you describe your practice?**
**( please skip this question if you answered NO in the previous question)**

A. Integral part of my dental practice
B. Regular bases
C. Occasionally
D. Not often

**19. If yes, please select additive manufacturing application from below:**
**(please skip this question if you answered NO in question 17)**

A. Diagnostic casts and models
B. Fixed prosthodontics
C. Removable prosthodontics
D. Surgical guides for implant placement
E. Radiographic stents
F. Occlusal appliances
G. Orthodontic aligners
H. Anatomical models for pre-surgical assessment, planning and training
I. Maxillofacial surgery
J. Scaffold for tissue engineering
K. Other

**20. If you have previously worked with additive manufacturing, what type of material have you used?**

A. Resin
B. Metal
C. Ceramics
D. Haven’t worked with additive manufacturing yet

**21. Does the curriculum of your university cover additive manufacturing in dentistry?**

A. Yes
B. No

**22. If yes, how would you describe it**
**( please skip this question if you answered NO in the previous question)**

A. Overly covered
B. Well covered
C. Sufficiently covered
D. Needs improvement

**23. If no, is there any plans to incorporate it in the curriculum during the next 2 years? ( please skip this question if you answered YES in question 21)**

A. Yes
B. No
